# Further Treatment Intensification in Undifferentiated and Rheumatoid Arthritis Patients Already in Low Disease Activity has Limited Benefit towards Physical Functioning

**DOI:** 10.1186/s13075-017-1425-7

**Published:** 2017-09-30

**Authors:** Sytske Anne Bergstra, Otto Olivas, Gülşah Akdemir, Naghmeh Riyazi, Gerard Collée, Johannes H. L. M. van Groenendael, Robert B. M. Landewé, Cornelia F. Allaart

**Affiliations:** 1 Department of Rheumatology, Leiden University Medical Centre, C1-R, Postbox 9600, 2300 RC Leiden, The Netherlands; 2 Hospital Universitario 12 de Octubre, Madrid, Spain; 3 Medical Centre Haaglanden, The Hague, The Netherlands; 4 Reumazorg Southwest Netherlands, Bergen op Zoom, The Netherlands; 5 Amsterdam Rheumatology & Immunology Centre and Zuyderland Medical Centre Heerlen, Heerlen, The Netherlands

**Keywords:** Early rheumatoid arthritis, Low disease activity, Physical functioning, Treatment

## Abstract

**Background:**

It is recommended to optimise treatment as long as a predefined treatment target is not met, but should the aim be remission if patients are in low disease activity (LDA)? The aim of this study was to assess if, in patients with rheumatoid arthritis (RA) or patients with undifferentiated arthritis (UA) with Disease Activity Score (DAS) ≤ 2.4 (LDA), treatment intensification results in better functional ability.

**Methods:**

In the IMPROVED study 610 patients with early RA or UA were treated with methotrexate + tapered high-dose prednisone. After 4 months, patients with DAS ≥ 1.6 were randomised to either of two treatment strategies. Patients with DAS < 1.6 tapered treatment. Over 5 years, patients with DAS ≥ 1.6 required treatment intensification, but protocol violations occurred, which allowed us to test the effect of treatment intensification regardless of subsequent DAS. A linear mixed model was used to test, in patients in LDA, the relationship between treatment intensification and functional ability (Health Assessment Questionnaire [HAQ]) over time.

**Results:**

The number of patients in LDA per visit ranged from 88 to 146. Per visit, 27–74% of the patients in LDA had treatment intensification. We found a statistically significant effect of treatment intensification on ΔHAQ, corrected for baseline HAQ, age, sex and treatment strategy (β = −0.085, 95% CI −0.13 to −0.044). When ΔDAS was added, the effect of treatment intensification was partly explained by ΔDAS, and the association with HAQ was no longer statistically significant (β = −0.022, 95% CI −0.060 to 0.016). When the interaction between treatment intensification and time in follow-up was added, a statistically significant interaction was found (β = 0.0098, 95% CI 0.0010 to 0.019), indicating lesser improvement in HAQ after treatment intensification if follow-up time increased.

**Conclusions:**

For patients with early RA and patients with UA already in LDA, further treatment intensification aimed at DAS remission does not result in meaningful functional improvement.

**Trial registration:**

ISRCTN, 11916566. Registered on 28 December 2006. EudraCT, 2006-006186-16. Registered on 16 July 2007.

**Electronic supplementary material:**

The online version of this article (doi:10.1186/s13075-017-1425-7) contains supplementary material, which is available to authorized users.

## Background

In the past few decades, the treatment of rheumatoid arthritis (RA) has changed considerably. Earlier treatment with disease-modifying anti-rheumatic drugs (DMARDs) has resulted in a milder disease course with better functional ability, as measured, for example, by the Health Assessment Questionnaire (HAQ) [1], and with less joint damage progression [2, 3]. One of the main aims of RA treatment is to achieve or maintain good physical functioning. To achieve this, it is recommended to start treatment early and regularly monitor disease activity and optimise treatment as long as a predefined treatment target has not yet been achieved (the ‘treat-to-target’ approach) [4]. International recommendations state that at least low disease activity (LDA; e.g., Disease Activity Score [DAS] ≤ 2.4), but preferably remission (e.g., DAS ≤ 1.6 or more stringent definitions), is the best treatment target when treating patients with RA [5]. Previous research has shown that a patient’s functional ability is related to DAS and, after prolonged disease activity, also to joint damage [6–9]. Moreover, a stronger decrease in DAS is associated with a stronger decrease in HAQ, even if DAS is already low [10]. However, it may be a patient characteristic rather than a further treatment intensification that determines how low a DAS and HAQ can be achieved. It has never been proved that intensifying drug therapy in patients who are already in LDA will result in further improvement in functional ability that is clinically meaningful. Because treatment intensification may not always be effective in further lowering disease activity and may come with potential side effects and costs, it is worthwhile to test the effect on functional ability of the effort itself, independent of the subsequent observed DAS outcome. In the present study we assessed whether aiming for remission and modifying or intensifying treatment accordingly in patients who are already in LDA results in further clinically relevant improvements in functional ability, regardless of a subsequent change in DAS.

## Methods

### Study design

The present study was an observational secondary analysis of data from the IMPROVED study. For this study, visits of patients in LDA (DAS > 1.6 but ≤ 2.4) were selected at each time point of the original study, and the effect of treatment intensification versus no treatment intensification on the change in HAQ observed at the next visit was analysed.

The IMPROVED study is a multicentre, randomised, single-blind, two-step clinical trial in patients with recent-onset RA and patients with undifferentiated arthritis (UA). Patients were recruited between March 2007 and September 2010 from 12 hospitals in the western part of The Netherlands. Recent-onset RA was diagnosed according to the 2010 American College of Rheumatology/European League Against Rheumatism (ACR/EULAR) classification criteria, with symptom duration ≤ 2 years [11]. UA was defined as arthritis in at least one joint and at least one other painful joint clinically suspected by the rheumatologist to be early RA but not fulfilling the 2010 criteria. The study protocol was approved by the medical ethics committee of each participating centre, and all patients gave written informed consent. A detailed description of the study has been reported previously [12].

Patients were ‘treated to target’, aimed at DAS remission (DAS in 44/53 joints < 1.6), with assessment of disease activity every 4 months during a 5-year period. Treatment was tapered and discontinued if DAS remission was achieved and henceforth maintained. Treatment was restarted, changed or intensified (henceforth called *treatment intensification*) if DAS remission was not achieved or was lost. The protocol required all patients to have started induction therapy with methotrexate 25 mg/week for 4 months and a tapered high dose of prednisone starting with 60 mg/day and tapered to 7.5 mg/day in 7 weeks. For patients in early DAS remission (DAS < 1.6 after 4 months), prednisone was tapered to 0, and if DAS remission persisted after 8 months, methotrexate was also tapered to 0. If DAS was ≥ 1.6 after 8 months, prednisone was restarted at 7.5 mg/day. In case of DAS ≥ 1.6 after restarting prednisone, patients were randomised (‘delayed randomisation’) to arm 1 or arm 2. Patients not in early DAS remission were randomised either (1) to methotrexate 25 mg/week + hydroxychloroquine 400 mg/day + sulphasalazine 2000 mg/day + prednisone 7.5 mg/day (arm 1) or (2) to a combination of adalimumab 40 mg/2 weeks + methotrexate 25 mg/week (arm 2). When patients did not achieve DAS remission at 8 months, those in arm 1 were switched to adalimumab + methotrexate, and for those in arm 2, the dosage of adalimumab was increased to 40 mg/week. For patients in both arms who achieved DAS remission within 8 months, treatment was tapered to methotrexate monotherapy. If patients in both groups did not achieve DAS remission with adalimumab 40 mg/week, further treatment was left to the opinion of the treating rheumatologist.

During the follow-up of the IMPROVED study, several protocol violations occurred and were monitored every 4 months. If treatment was not intensified in patients who were in LDA, this was registered as a protocol violation. In the present study, we compared subsequent changes in functional ability for patients in LDA (DAS > 1.6 but ≤ 2.4) who did or did not have a protocol violation (no treatment intensification versus treatment intensification), which allowed us to investigate the effect of treatment intensification on HAQ change.

### Statistical analysis

Functional ability was measured every 4 months using the Dutch version of the HAQ [13]. A change in HAQ score ≥ 0.22 in a patient is considered clinically relevant [14]. At each time point, all visits where patients were in LDA (DAS ≤ 2.4 but > 1.6) were selected. Thus, the number of included visits per patient could differ. Visits of patients in LDA with treatment intensification (according to protocol) and without treatment intensification (protocol violation) were compared. Differences in HAQ and DAS at each visit compared with the next visit were calculated [ΔHAQ and ΔDAS; i.e., *Y*(*t* + 1) − *Y*(*t*)], and a negative ΔHAQ or ΔDAS implied improvement. Linear mixed model analyses with random intercepts were performed to test the relationship between treatment intensification and ΔHAQ over time, taking into account the correlation of visits within a patient. Models were fitted using restricted maximum likelihood. For each model, we tested whether allowing a random slope improved the fit of the model. If not, we tested which covariance matrix for within-cluster residuals gave the best fit of the model. Three models were fitted, and each model was adjusted for the possible confounders follow-up time, baseline HAQ, age, sex and treatment arm. In the second model, the effect of ΔDAS on the model also was tested. In the third model, the interaction effect between change in treatment and follow-up time was added. All analyses were performed using STATA SE version 14 software (StataCorp, College Station, TX, USA).

## Results

Over a period of 5 years, both DAS and HAQ showed statistically significant improvement across all patients included in the original study (mean [SD] baseline HAQ 1.2 [0.7], ΔHAQ −0.59, 95% CI −0.61 to −0.57; mean [SD] baseline DAS 3.2 [0.9], ΔDAS −1.77, 95% CI −1.79 to −1.75]. In 69% of the patients, the change in HAQ was clinically meaningful (≥0.22).

The number of patients in LDA ranged from 88 to 146 per visit, of which 26–73% did not get treatment intensification, with an increase in such protocol violations towards the end of the study (Additional file 1). In total, 482 patients were in LDA at one or more visits where there was information available regarding medication use as well as a follow-up visit, resulting in a total number of 1532 visits available for analysis. The average patient and disease characteristics for all included visits where patients were in LDA are provided in Table 1. Patients with a treatment intensification more often fulfilled the ACR/EULAR 2010 criteria and were more often male and rheumatoid factor- and anti-citrullinated protein antibody-positive, although most differences were small.

**Table 1:** Average patient and disease characteristics for all included visits with Disease Activity Score ≤ 2.4 but > 1.6

	No treatment intensification	Treatment intensification
Age, years, mean (SD)	52.6 (12.6)	51.0 (12.4)
Sex, *n* (% female)	46 (78.9)	39 (68.4)
Treatment arm		
Early remission	46.2	57.2
MTX + SSZ + HCQ + prednisone	20.9	19.9
MTX + adalimumab	19.1	16.0
Out of protocol	13.8	6.7
Symptom duration, weeks, median (IQR)	20 (9–35)	19 (9–32)
Diagnosed RA, % meeting 2010 ACR/EULAR criteria	46 (79.2)	47 (84.5)
Anti-citrullinated protein antibodies, % positive	34 (57.6)	35 (61.9)
Rheumatoid factor, % positive	33 (58.9)	34 (63.0)
Health Assessment Questionnaire (0–3)^a^, mean (SD)	0.78 (0.56)	0.63 (0.48)
Disease Activity Score, mean (SD)	1.95 (0.23)	1.99 (0.23)
Tender joint count, median (IQR)	2 (2–4)	3 (2–4)
Swollen joint count, median (IQR)	0 (0–1)	1 (0–2)
VAS general health (0–100)^b^, mean (SD)	31.0 (19.6)	31.7 (20.3)
Erythrocyte sedimentation rate, mm/h, median (IQR)	15.7 (13.0)	13.9 (11.2)

*Abbreviations: ACR/EULAR* American College of Rheumatology/European League Against Rheumatism, *RA* Rheumatoid arthritis, *VAS* Visual analogue scale, *MTX* Methotrexate, *SSZ* Sulphasalazine, *HCQ* Hydroxychloroquine

The average number of patients per visit with low disease activity without a treatment intensification was 56 (range 24–103), and the average number of patients per visit with low disease activity with treatment intensification was 61 (range 30–77)

^a^0 = No functional limitations

^b^100 = Best score

For patients in LDA, after treatment intensification the mean (SD) change in DAS at the next visit was −0.48 (0.71), resulting in remission in 59% of the visits. In cases where there was no treatment intensification, this was −0.15 (0.67), resulting in remission in 38% of the visits. The mean (SD) changes in HAQ at the next visit for patients in LDA were −0.083 (0.37) after treatment intensification, resulting in a clinically meaningful change in HAQ in 24% of the visits, and −0.0011 (0.35) without treatment intensification, resulting in a clinically meaningful change in HAQ in 25% of the visits.

Results of the linear mixed model analyses to assess the effect of treatment intensification on ΔHAQ are shown in Table 2. All models had a random intercept and an independent covariance matrix. We found a small but statistically significant effect of treatment intensification on ΔHAQ, corrected for baseline HAQ, time in follow-up, age, sex and treatment arm (model 1 β = −0.085, 95% CI −0.13 to −0.044). The unadjusted model showed a larger effect (β = −0.12, 95% CI −0.15 to −0.08). This points to a weak association between treatment intensification and an improvement in HAQ: Patients with a treatment intensification had a 0.085 additional improvement in ΔHAQ over time compared with patients without treatment intensification. When ΔDAS was added (model 2), the association between treatment intensification and ΔHAQ became weaker and was no longer statistically significant (β = −0.022, 95% CI −0.060 to 0.016). Patients with treatment intensification now had only a 0.022 additional improvement in ΔHAQ over time compared with patients without treatment intensification. When the interaction between treatment intensification and time in follow-up was subsequently added (model 3), a statistically significant interaction was found (β = 0.0098, 95% CI 0.0010 to 0.019), suggesting that the association between treatment intensification and HAQ improvement, already weak in the early phases, only becomes weaker over time. Again, the unadjusted model showed a larger effect (β for treatment intensification = −0.24, 95% CI −0.32 to −0.15; β for time = −0.005, 95% CI −0.012 to 0.0027; β for treatment intensification × time = 0.017, 95% CI 0.0075 to 0.027).

**Table 2:** Linear mixed model analyses to assess effect of treatment intensification on change in Health Assessment Questionnaire

	β	95% CI	*p* Value
Model 1 (*n* patients = 479, *n* visits = 1528)			
Treatment intensification	−0.085	−0.13 to −0.044	< 0.001
Follow-up time^a^	0.0057	0.00094 to 0.010	0.019
Model 2 (*n* patients = 476, *n* visits = 1509)			
Treatment intensification	−0.022	−0.060 to 0.016	0.246
Follow-up time^a^	0.0022	−0.0021 to 0.0066	0.313
DAS change	0.23	0.21 to 0.26	< 0.001
Model 3 (*n* patients = 476, *n* visits = 1509)			
Treatment intensification	−0.10	−0.18 to −0.021	0.013
Follow-up time^a^	−0.0034	−0.010 to 0.0033	0.323
Treatment intensification × follow-up time	0.0098	0.0010 to 0.019	0.029
DAS change	0.23	0.21 to 0.26	< 0.001

*DAS* Disease Activity Score in 44/53 joints

^a^Follow-up time is added to the model as visit number, with time between visits being 4 months. All models were adjusted for baseline Health Assessment Questionnaire, sex, age and treatment arm

## Discussion

In this observational secondary analysis of data from a randomised clinical trial, we assessed whether intensifying drug therapy in patients who are in LDA but not in remission results in a clinically meaningful improvement in physical functioning as measured by the HAQ. We found that intensifying treatment in patients with RA or patients with UA in LDA resulted in a statistically significant improvement in ΔHAQ over time. However, the effect was rather small and appears clinically irrelevant. The improvement in ΔHAQ was partly explained by ΔDAS, and the effect of treatment intensification or change on ΔHAQ decreased by increasing follow-up time.

It is currently recommended that treatment efforts in patients with RA be aimed at remission or LDA [15]. The question remains whether patients would further benefit from a treatment aimed at remission if they are already in LDA. Several studies have already confirmed the relationship between ΔDAS and ΔHAQ, also with longer follow-up [6–8, 10]; however, those studies aimed for LDA and/or assessed the relationship between ΔDAS and ΔHAQ in a cross-sectional manner. Previous research also showed that patients with sustained clinical remission (≥ 24 weeks) had a continuous improvement in HAQ values and that remission implies better physical functioning than LDA [16–18]. However, finding that some patients achieved remission and had lower HAQ than the patients who did not achieve remission may have been coincidental and not the result of a therapeutic intervention, because none of these studies assessed prospectively whether further aiming for remission by intensifying treatment in patients who already have achieved LDA results in further clinically relevant improvement in HAQ. The IMPROVED study provided the opportunity to test this because the study protocol formally required treatment intensification as long as DAS was not < 1.6. However, rheumatologists did not always comply with this formal requirement, thus allowing us to compare outcomes after treatment intensification vs. lack thereof in patients with DAS < 2.4 but still > 1.6. In addition, we could investigate if such an association was dependent on the time of follow-up.

Our results suggest that the minimally positive effect of a treatment intensification on ΔHAQ is present mainly at the start of treatment and that it decreases by increasing treatment duration. This observation is in line with earlier findings and current guidelines that patients with RA should be treated early in the disease process [4, 19, 20]. It also suggests that in patients with early RA and patients with UA, initial treatment should consist of (a combination of) highly effective drugs to decrease disease activity rapidly and thus maximally improve physical functioning. Persistently aiming for remission in patients already in LDA may lead to inappropriate treatment intensification and increased use of anti-rheumatic drugs (overtreatment), without additional benefits. This was recently found in studies where clinical remission and imaging-based remission were compared as treatment targets [21, 22].

A limitation of this study was that we looked only at treatment intensification in general and did not specify the type of treatment changes. Different treatments may have different effects on physical functioning. A second limitation of our analysis is that patients with LDA in whom treatment was intensified may have differed from those for whom treatment was not intensified with respect to characteristics that are relevant to the outcome of interest but that we did not measure (intangible confounders). Previous studies also showed not only that ΔHAQ is associated with ΔDAS but also that an increase in joint damage may lead to worse physical functioning, especially with longer follow-up [6–9]. Because in the remission-steered IMPROVED study the majority of the patients hardly had any radiographic damage, joint damage was not further considered in this analysis [23].

## Conclusions

Treatment intensification in patients with early RA or patients with UA who have already achieved LDA is associated with a statistically significant decrease in HAQ, but not with a clinically meaningful improvement in functional ability, during 5 years of DAS remission-steered treatment. Therefore, not remission or LDA but good functional ability may be the optimal treatment target at which to steer treatment adjustments. Thus, it might be sufficient to accept achieved LDA rather than continue treatment intensification aimed at remission. Further treatment intensification may not lead to a clinically relevant improvement in HAQ, but it may have downsides, such as side effects and costs.

## Additional file

Additional file 1 Number of patients in low disease activity with or without protocol violation for each visit. (DOCX 210 kb)

## Abbreviations

ACR/EULAR: American College of Rheumatology/European League Against Rheumatism
DAS: Disease Activity Score
DMARD: Disease-modifying anti-rheumatic drug
HAQ: Health Assessment Questionnaire
HCQ: Hydroxychloroquine
LDA: Low disease activity
MTX: Methotrexate
RA: Rheumatoid arthritis
SSZ: Sulphasalazine
UA: Undifferentiated arthritis
VAS: Visual analogue scale
