# Supplementary material for: Further Treatment Intensification in Undifferentiated and Rheumatoid Arthritis Patients Already in Low Disease Activity has Limited Benefit towards Physical Functioning
Source: Arthritis Res Ther. 2017 Sep 30;19:220. doi: 10.1186/s13075-017-1425-7 (PMC5622576; doi:10.1186/s13075-017-1425-7)
Supplement: Additional file 1 — Number of patients in low disease activity with or without protocol violation for each visit. (DOCX 210 kb) [file 13075_2017_1425_MOESM1_ESM.docx]

**Additional file 1: Number of patients in low disease activity with or without protocol violation for each visit**

Additional figure 1. Number of visits with patients in low disease activity with (dark blue) or without protocol violation (light blue) for each visit.
